# Comparison of different sepsis scoring systems and pathways: qSOFA, SIRS, Shapiro criteria and CEC SEPSIS KILLS pathway in bacteraemic and non-bacteraemic patients presenting to the emergency department

**DOI:** 10.1186/s12879-022-07070-6

**Published:** 2022-01-22

**Authors:** Rebecca Sparks, Arisa Harada, Ruchir Chavada, Christopher Trethewy

**Affiliations:** 1 Department of Microbiology and Infectious Diseases, NSW Health Pathology-Central Coast, Gosford, Australia; 2 Department of Medicine, Gosford Hospital, Gosford, Australia; 3 Emergency Department, Gosford Hospital, Gosford, Australia

**Keywords:** Bacteraemia, Sepsis, Sepsis scoring systems, Sepsis in emergency department

## Abstract

**Background:**

Bacteraemia is associated with high morbidity and mortality, with delayed antibiotic treatment associated with poorer outcomes. Early identification is challenging, but clinically important. Multiple scoring systems have been developed to identify individuals in the broader categories of sepsis. We designed this study to assess the performance of existing scoring systems and pathways—CEC SEPSIS KILLS pathway (an Australian sepsis care package), quick sequential organ failure score (qSOFA), systemic inflammatory response syndrome (SIRS) and the Shapiro criteria.

**Methods:**

This was a retrospective cohort study performed in two metropolitan hospitals in NSW, consisting of adult patients (> 18 years) with positive blood cultures containing a true pathogen and patients matched by age without positive blood cultures. Performance (sensitivity, specificity, and mortality prediction) of recognised sepsis and bacteraemia criteria and pathways—qSOFA, SIRS, Shapiro criteria and CEC SEPSIS KILLS pathway in the first 4 h following ED triage was assessed.

**Results:**

There were 251 patients in each cohort. Sepsis-related mortality was higher in the bacteraemic group (OR 0.4, p = 0.03). Of the criteria studied, the modified Shapiro criteria had the highest sensitivity (88%) with modest specificity (37.85%), and qSOFA had the highest specificity (83.67%) with poor sensitivity (19.82%). SIRS had reasonable sensitivity (82.07%), with poor sensitivity (20.72%). The CEC SEPSIS pathway sensitivity of 70.1% and specificity of 71.1%. The SEPSIS KILLS was activated on only 14% of bacteraemic patients.

**Conclusion:**

The performance of all scoring systems and pathways was suboptimal in the identification of patients at risk of bacteraemia presenting to the emergency department.

**Supplementary Information:**

The online version contains supplementary material available at 10.1186/s12879-022-07070-6.

## Background

Sepsis a heterogeneous syndrome broadly defined as life-threatening organ dysfunction caused by a deregulated host response to infection [1]. It remains a leading cause of mortality in patients at extremities of age, the immunosuppressed and individuals with multiple comorbidities, while higher mortality rates have been observed in individuals with bacteraemia associated with sepsis than those without [2–4]. Survival benefit has been linked to prompt identification of sepsis and septic shock and early administration of antimicrobials, leading to the development of global initiatives including the Surviving Sepsis campaign (developed by the Society of Critical Care Medicine and the European Society of Intensive Care) which provides management plans based on these principles [5, 6]. Locally the Australian Commission on Safety and Quality in Healthcare are in the consultation stage of developing a national sepsis program.

In 1992, the American College of Chest Physicians (ACCP) and the Society of Critical Care Medicine (SCCM) introduced a definition for the Systemic Inflammatory Response Syndrome (SIRS), defining the early response of the host to a nonspecific insult, which may be infectious. Review of this criteria found it to have poor sensitivity in critically ill patients, and failed to distinguish between a host inflammatory response due to infective or and non-infective causes, limiting its use in identifying those at risk of sepsis [7, 8]. In 2008, Shapiro et al. developed a clinical prediction rule to stratify patients at risk of developing bacteraemia presenting to the Emergency Department (ED), in an attempt to rationalise blood culture procurement whilst identifying those at high risk of bacteraemia [9]. The criteria had subsequently been found to have reasonable sensitivity but poor specificity [10]. In 2016, SEPSIS-3 introduced the quick Sequential [Sepsis Related] Organ Failure score (qSOFA) to identify patients at high risk of mortality from sepsis presenting to the ED, due to its greater prognostic accuracy for in-hospital mortality [11]. Based on the simplicity of the score, the Third International Consensus Definitions for Sepsis recommended qSOFA as a prompt, accurate means of identifying patients who were likely to be septic. However, like other scoring systems mentioned above this score had low sensitivity identifying patients at risk of sepsis [12].

On a local level, in 2011 NSW Clinical Excellence Commission (CEC) introduced a state-wide SEPSIS KILLS pathway in all EDs. This pathway incorporated bundles of care that included taking blood cultures, antibiotic administration within an hour of triage and fluid resuscitation. This program aimed to reduce preventable harm to patients through improving the recognition and management of sepsis. For early visual recognition in ED, SEPSIS kills icon was created with an aim for its use by the ED triage nurse. The CEC pathway was based on the SEPSIS-2 definition of sepsis, which involved infection in the presence of 2 systemic inflammatory response syndrome (SIRS) criteria, substituting local ‘Between the Flags’ parameters [13]. Overall mortality appeared to decrease with the implementation of SEPSIS KILLS in NSW hospitals [14].

Considering the high mortality associated with bacteraemia in the septic patient, the primary outcome of this study was to determine the sensitivity and specificity of various sepsis criteria in the existing literature within a cohort of confirmed bacteraemic patients, when compared to an age matched non bacteraemic population.

## Methods

### Study setting, population and study design

This retrospective, age-matched cohort study was conducted over two acute, mixed metropolitan EDs located on the Central Coast of NSW, Australia. Collectively the departments have an annual census of 130,000 presentations and have a total inpatient bed capacity of 750.

Adult patients (> 18 years) who had blood cultures taken during their ED workup were randomly selected from a blood culture database between January 1, 2016, and December 31, 2017. Patients with positive cultures thought secondary to sample contamination (determined either by a microbiologist or the medical care provider) were excluded from the study. This included coagulase-negative staphylococcus, *Cutibacterium spp., Bacillus spp* and *Corynebacterium spp*. Two-hundred and fifty-one patients with true positive blood cultures were included in the study, with a further age-matched cohort of 251 patients with negative blood culture included from the database. In total, 502 patients were included in the study.

**Fig. 1:**
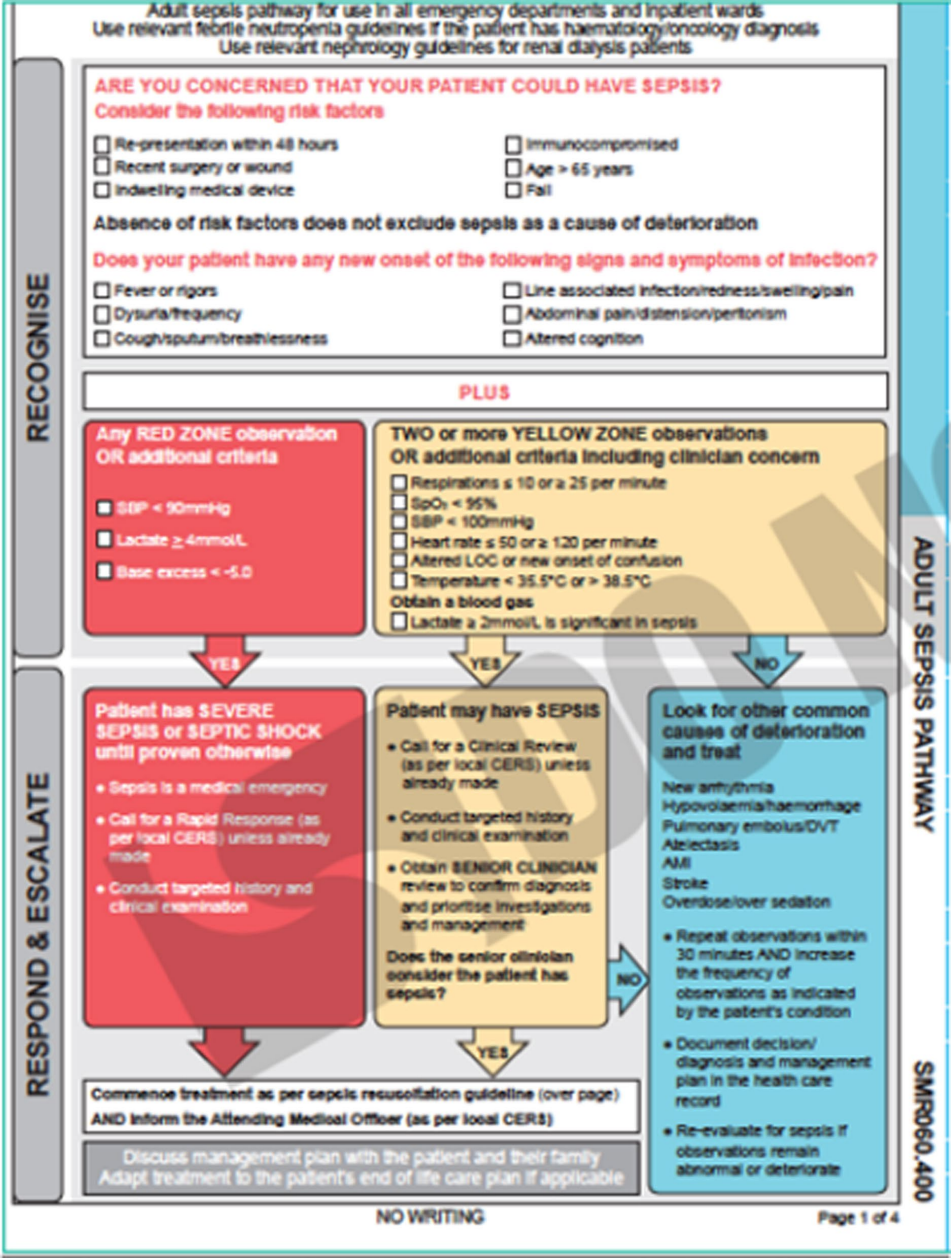
CEC SEPSIS KILLS pathway

A single unblinded reviewer collected data retrospectively from electronic medical records and paper files using standardised data collection sheet including basic demographics, time of triage and discharge, and whether antibiotic therapy was given within 1 h of triage. Secondary outcomes including transfer to and length of stay in ICU, total hospital length of stay, in-hospital mortality, all-cause and due to sepsis, were recorded. Vital signs documented at triage, laboratory results and initial presenting symptoms within the first four hours of triage were used to determine whether SIRS, qSOFA, Shapiro or CEC SEPSIS KILLS criteria were triggered in each episode (Table 1, Fig. 1, Additional file 1). White cell bands were not routinely reported by the local haematology laboratory, and therefore not included in criteria calculations (SIRS and Shapiro). A sepsis-related death (Sepsis mortality) was defined by the declaration of the cause of death due to an infective process as documented on the death certificate by the treating clinician.

**Table 1:** Definition of Scroing Systems

Scoring system	SIRS	Quick Sequential Organ Failure Score (qSOFA)	Modified Shapiro Criteria
Parameters	1. Temperature > 38 or < 36 °C 2. Respiratory rate > 20 breaths per minute 3. Heart rate > 90 beats per minute 4. White cell count > 12 or < 4 or bands > 10%	1. Altered mental state 2. Respiratory rate ≥ 22 breaths per minute 3. Systolic blood pressure ≤ 100 mmHg	Major criteria 1. Suspected endocarditis, 2. Temperature ≥ 39.4 °C 3. Indwelling vascular advice Minor criteria 1. Age > 65 2. Temperature 38.3–39 3. Chills 4. Vomiting 5. Systolic blood pressure ≤ 90 mmHg 6. White cell count > 18 7. Bands > 5% 8. Platelets < 150 9. Creatinine > 200 mg/dL
	Score ≥ 2 = meets criteria	Score ≥ 2 = meets criteria	≥ 1 major or ≥ 2 minor = meets criteria

### Statistical analysis

Categorical variables were assessed using Chi-squared test and continuous variables using Mann–Whitney t-test as the data was hypothesised to be non-parametric. Standard univariate analysis was performed between the two groups. The Receiver Operating Characteristics (ROC) of each of the scores and the CEC pathway were plotted to define the diagnostic ability and discrimination threshold. Area under the ROC curve, sensitivity and specificity and confidence intervals were calculated. The interrater precision of the data was quantified using a Cohen’s Kappa analysis, comparing a random selection of 5% of the cohort data, extracted by a second, blinded observer (CT), using the same data extraction tool.

Descriptive analysis was performed using GraphPad Software (Prism 7.0 for Mac OSX, GraphPad, CA) and SPSS 26 (IBM, Armonk, NY): IBM Corp.

## Results

The strength of agreement between the observers was considered very good (K = 0.983, 95% CI 0.969–0.998).

### Demographic, and secondary outcome data

There were 18,469 blood cultures taken in the ED at Gosford and Wyong Hospital from 2016 to 2017, including both paediatric and cases, with 2139 positive cultures (11.6%), including true pathogens and contaminated samples.

When comparing Bacteraemia and non-Bacteraemia cohorts, there was no difference between the groups in terms of mean age or gender. The proportion of patients admitted to the ICU (10.7% vs 9.2%; p = 0.11) was not statistically significant, nor was the mean length of stay (4.4 versus 6.7 days; p 0.11). Bacteraemia was significantly associated with longer duration of antibiotic therapy (15.44 days vs 8.20; p < 0.001) and a longer length of inpatient admission (10.73 days vs 6.06 days; p < 0.001). When the mortality was compared between the bacteraemic and non-bacteraemic groups, sepsis related mortality was proportionately significant in the bacteraemic group (p 0.03) (Table 2).

**Table 2:** Demographic and comparative hospital data

	Bacteraemic (n = 251)	Non bacteraemic (n = 251)	p value	Odds ratio	95% CI
Age (year) (range)	72.06 (21–96)	72.07 (23–96)	0.99	–	–
Gender M (%)	131 (52.2)	14 (56.1)	0.37	–	–
ICU admission (%)	27 (10.7)	23 (9.2)	0.55	–	–
ICU LOS days	4.4	6.7	0.11	–	− 0.53–5.10
Hospital LOS; days (range)	10.7 (0–64.8)	7.5 (0.98.6)	< 0.01	–	− 5.27 to − 1.28
Duration of antibiotics (range)	15.44 (0–153)	8.2 (0–68)	< 0.01	–	− 9.43 to − 5.04
Mortality sepsis (%)	19 (7.5)	8 (3.1)	0.03	0.4	0.17–0.94
Mortality hospital (%)	21 (8.4%)	11 (4.4%)	0.068	2.0	0.94–4.2

**Fig. 2:**
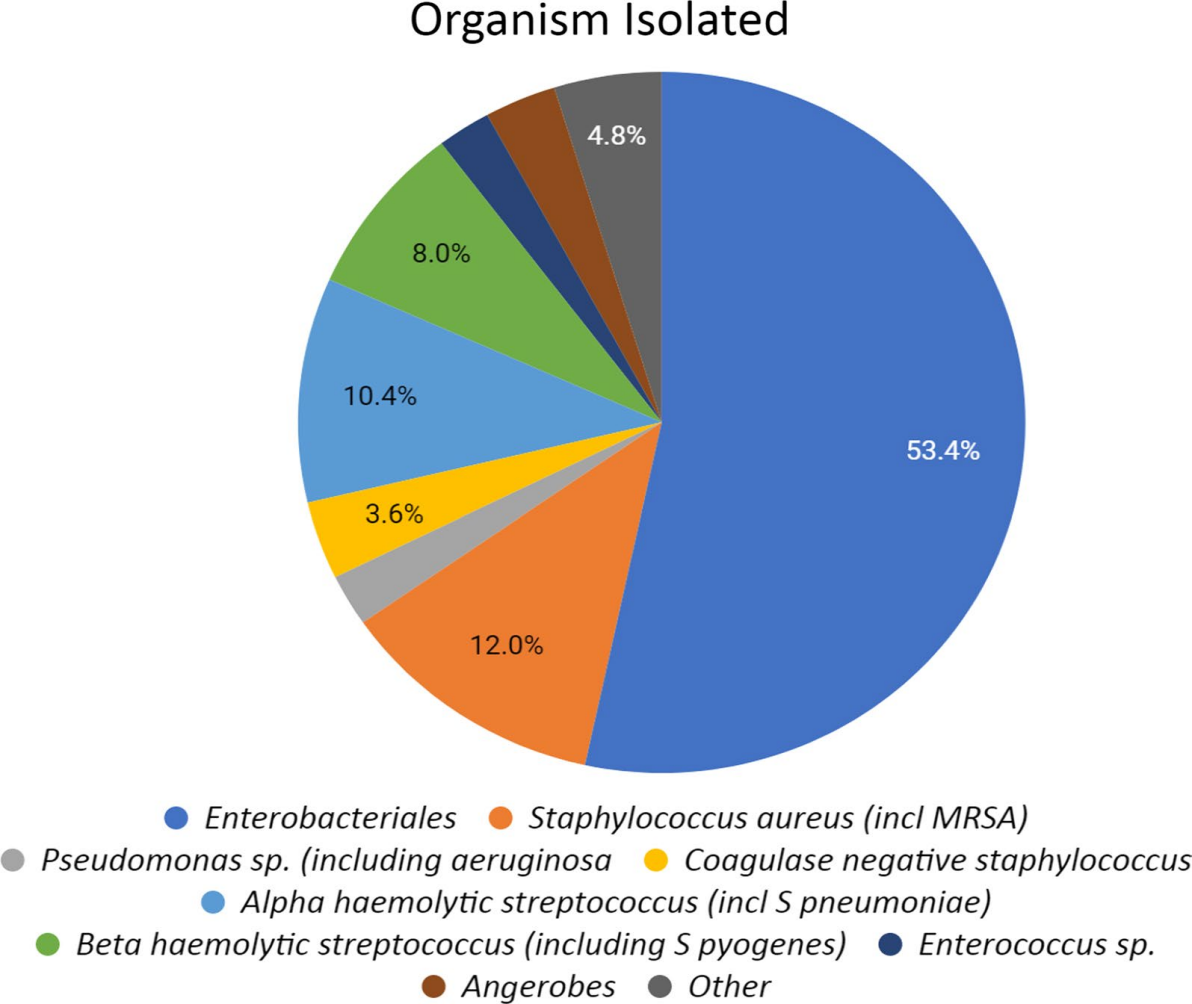
Infectious organism causing invasive disease in 251 patients with bacteraemia in the ED

Source of infection differed in the two groups, with most common source in the bacteraemic patients was unknown, while the non bacteraemic had a predominance of respiratory cases. Mortality was highest in the respiratory cohort of both bacteraemic (32%) and non bacteraemic (62%) populations (Table 3). The most common pathogen detected in blood cultures was Enterobacteriales (53%), followed by Staphylococcus aureus (including MRSA) (12%) (Fig. 2).

**Table 3:** Likely source of sepsis as per the ED physician conclusion of 251 bacteraemic and 251 non-bacteraemic patient

ED suspected source of infection	Bacteraemic		Non bacteraemic	
	Total n = 251 (%)	Sepsis mortality n = 19 (%)	Total n = 251 (%)	Sepsis mortality n = 8 (%)
Genitourinary	61 (24)	2 (10.5)	35 (14)	1 (12)
Respiratory	31 (12)	4 (21)	102 (41)	5 (62)
Skin/soft tissue/bone	28 (11)	2 (10.5)	37 (15)	1 (12)
Cardiac	7 (3)	2 (10.5)	1 (0.4)	0
Hepatobiliary	23 (10)	2 (10.5)	3 (1)	0
Gastrointestinal	22 (9)	1 (5)	22 (9)	0
Vascular disease	3 (1)	0	1 (0.4)	0
Central nervous system	6 (2)	0	9 (3)	0
Unknown	70 (28)	6 (32)	42 (17)	1 (12)

### Accuracy of mortality prediction

Of the bacteraemic cohort, 14% (34/251) were identified at Triage (SEPSIS KILLS on the eMR). When this was compared to the Non Bacteraemic cohort, proportionately more Bacteraemic patients were correctly identified. (p = 0.03). When the proportion of Bacteraemic patients receiving antibiotics within 1 h was compared with Bacteraemic patients who received antibiotics > 1 h after arrival in ED, in terms of identification at Triage using the SEPSIS KILLS icon, there was no statistically significant difference (p = 0.07). When the sepsis mortality rate of those identified at Triage with the SEPSIS KILLS icon was compared between the bacteraemic and non bacteraemic groups, there was no significant difference (p = 0.64). When the sepsis mortality rate of the bacteraemic group was compared with correct identification by the Triage SEPSIS KILLS icon there was no significant difference (p = 0.92). During the first four hours of admission within the ED, the SEPSIS KILLS pathway was utilised proportionately more times in the bacteraemic group when compared to the non bacteraemic group (179 versus 72; p < 0.01).

A positive Shapiro score identified 25/27 (92.5%) patients who subsequently died of sepsis. When the proportion of patients were compared in terms of Sepsis mortality and Shapiro criteria, proportionately more patients who fulfilled the Shapiro criteria had mortality attributed to Sepsis (OR 0.23 [95%CI 0.05 ≤ OR ≤ 0.98]; p = 0.03). The qSOFA score identified 7/27 (26%) of patients who died with sepsis-related cause and was positive a total of 91 times. When the proportion of patients were compared in terms of sepsis mortality and qSOFA score, proportionately more patients who fulfilled the qSOFA score died of Sepsis (OR 0.36 [95%CI 0.14 ≤ OR ≤ 0.94]; p 0.03). With regard to the SIRS criteria, when the proportion of patients who fulfilled the criteria was compared with the proportion of those who died of Sepsis, there was no significant difference between those patents with or without the features of SIRS. Proportionately more of the patients who died of Sepsis-related mortality were identified by the Shapiro criteria or qSOFA score in this study.

### Diagnostic accuracy of predictive scores

**Fig. 3:**
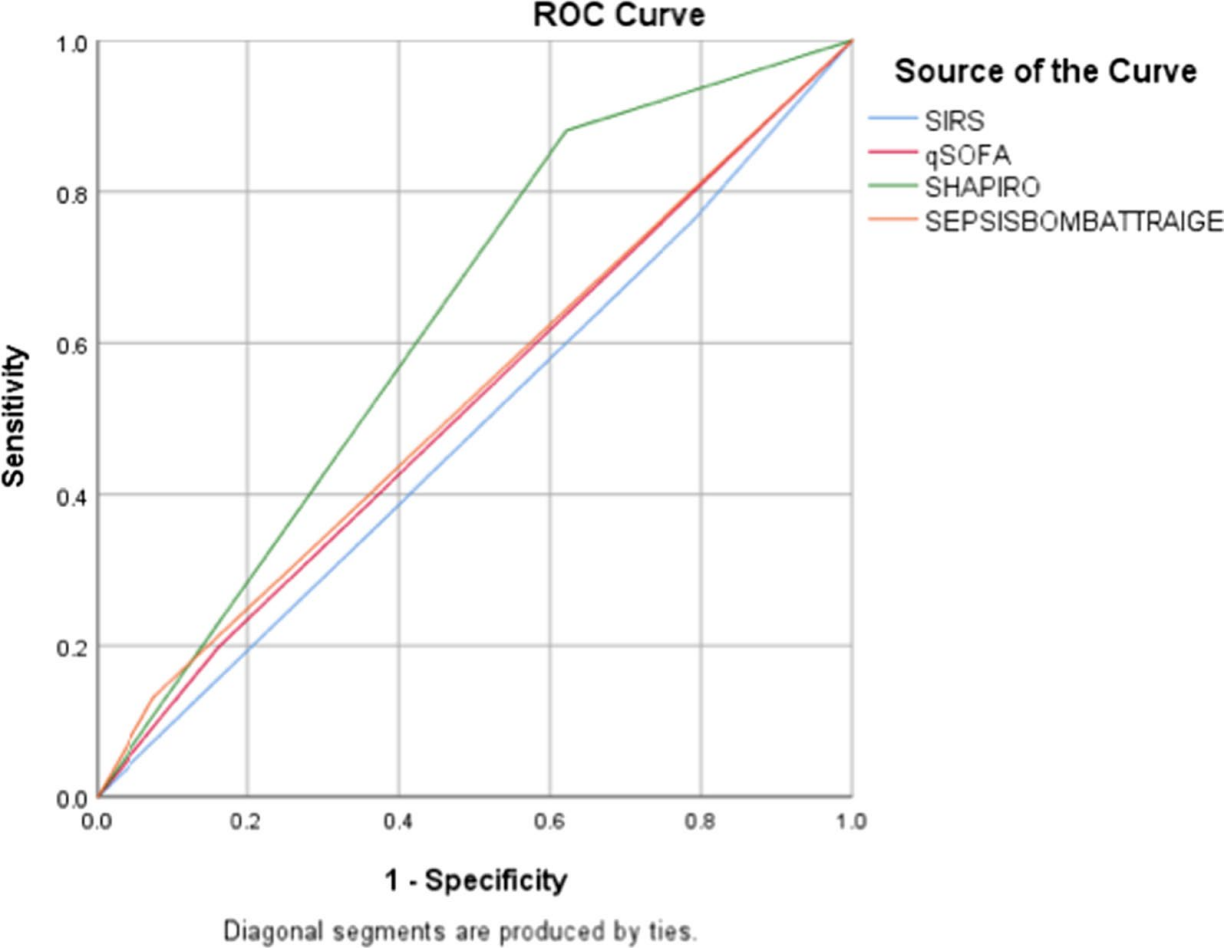
Receiver operating curve (ROC) of qSOFA, SIRS and Shapiro criteria in identifying bacteraemic patients

When the bacteraemic and non-bacteraemic cohort were compared using the three scoring systems, the modified Shapiro rule demonstrated the highest sensitivity of all tools assessed (88.05%), with modest specificity (37.85%) in detecting bacteraemia in the ED population. qSOFA was displayed the highest specificity (83.67%), however demonstrated poor sensitivity (19.82%). SIRS had reasonable sensitivity (82.07%), again with poor sensitivity (20.72%) (Table 4, Fig. 3). The area under ROC curve was 0.627 for the modified Shapiro rule, 0.517 for qSOFA and 0.514 for SIRS.

**Table 4:** Comparison of scoring system performance in predicting bacteraemia

Scoring system	Bacteraemic (n = 251)		Non bacteraemic (n = 251)		Area under ROC curve	Sensitivity	Specificity	Clinical predictive value
	Positive	Negative	Positive	Negative				
qSOFA	50	201	41	210	0.517 95% CI (0.4—0.56)	19.82 95% CI (15.16–25.4)	83.67 95% CI (78.50–88.02)	PPV 55% NPV 49%
SIRS	206	45	200	51	0.514 95% CI (0.46—0.56)	82.07 95% CI (76.76–86.61)	20.72 95% CI (15.88–26.26)	PPV 45% NPV 54%
SHAPIRO	221	30	154	95	0.627 95% CI (0.58–0.68)	88.05 95% CI (83.38–91.79)	37.85 95% CI (31.82–44.16	PPV 60% NPV 76%

**Fig. 4:**
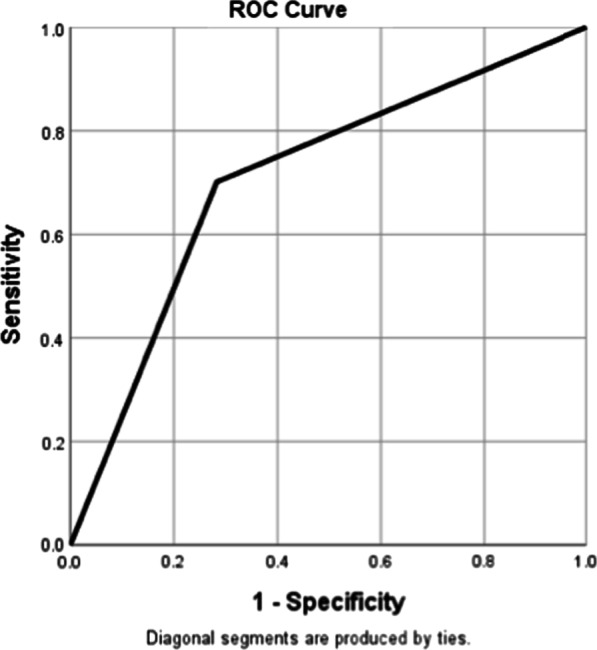
Receiver operating curve (ROC) of the CEC SEPSIS KILLS pathway in identifying bacteraemic patients

Evidence that during the first four hours of ED stay the treating Clinician commenced the SEPSIS KILLS pathway had both moderate sensitivity (69.6%) and specificity (71.31%) for Bacteraemic patients, with an area under the ROC curve 0.71 (Table 5, Fig. 4).

**Table 5:** Performance of CEC SEPSIS KILLS pathway in predicting bacteraemia

	Bacteraemic (n = 251)		Non bacteraemic (n = 251)		Area under ROC curve	Sensitivity	Specificity	Clinical predictive value
	Positive	Negative	Positive	Negative				
SEPSIS KILLS	175	76	72	179	0.71 95% CI (0.66–0.75)	69.72 95% CI (63.49–75.34)	71.31 95% CI 65.29–76)	PPV 71% NPV 70%

## Discussion

Overall in this study we found that the scoring systems and pathways assessed performed poorly, with low to moderate sensitivity and overall poor specificity. The positive predictive values were suboptimal in identifying bacteraemic patients presenting to the ED.

This study demonstrated that the modified Shapiro criterion was the most sensitive method of detecting true bacteraemia. However, our data suggests that this method is not sensitive enough as a stand-alone tool to predict bacteraemia, with almost 8% of patients whose bacteraemia would be missed using this scoring system alone. Our calculated sensitivity is lower than previous data, however has similar specificity. [10, 15, 16] Larger studies are therefore required to demonstrated reproducibility in these findings. Practical application of Shapiro in real-world settings should also be considered. Four of the nine minor criteria are obtained from haematological or biochemical blood analysis, meaning that the calculation of the score is not available at initial point of care. Waiting for these results may lead to delays in taking blood cultures and administering antibiotics in septic bacteraemic patients or result in blood cultures being taken after antibiotic administration, significantly reducing their yield. Prospective assessment of the score in real-time, with outcomes including delay to antibiotics and taking blood cultures should be examined.

The CEC SEPSIS KILLS pathway demonstrated the most balanced sensitivity and specificity in detecting bacteraemia with moderate accuracy. In initial development and application of the pathway, there was an emphasis on between the flags (BTF) vital sign criteria and serum lactate level only in the assessment of a patient with possible sepsis. However in recent years there has been the addition of other criteria including clinician clinical concern of sepsis. As the pathway now incorporates both vital signs and clinical assessment by a senior physician in determining the presence or absence of sepsis, it raises the possibility of inter-hospital variability of the tool due to variability in clinical experience. Another single centre NSW based study demonstrated low sensitivity (45.1%) and moderate specificity (78.9%) when clinical impression was not incorporated [17]. However other studies have shown an overestimation in bacteraemia prediction when based on clinical judgement alone [18]. Our study did not analyse whether the individual vital sign criteria was responsible for the activation of the SEPSIS KILLS pathway, or individual senior clinician suspicion, therefore we feel that a multisite assessment into this may be warranted to determine the performance of the tool with vital sign criteria alone. If the vital sign criteria alone were associated with reasonable sensitivity and specificity, we could feel more confident in this tool being used by clinicians of all skill levels.

While CEC SEPSIS KILLS criteria performed reasonably overall, the associated SEPSIS KILLS FirstNet tool was rarely applied in either group, with an activation rate of 14% in our patients with bacteraemia. We speculate that poor uptake by clinical staff was potentially due to inadequate of knowledge of the existence of the tool, the lack of clinical criteria provided in the CEC package in the recommendation for its application, inexperience and insufficient education of staff activating the tool and under-recognition of sepsis due to heterogeneity of presenting symptoms and its evolving pathophysiology. It is also clear that sepsis is an evolving process, so a single point of assessment at a variable point in the pathophysiological process is unlikely to be clinically reliable. We found that bacteraemic patients were correctly identified more often than non bacteraemic patients, which overall is of questionable significance given the overall poor rates of use of the tool. Identification of patients at risk of sepsis with the icon also did not lead to earlier administration of antibiotics. We recommend further evaluation of the uptake of the SEPSIS KILLS in other NSW EDs and include assessment of the rates of its use and clinical impact, including whether time to antibiotic therapy and fluid resuscitation were optimised. Targeted education programs could then be developed to inform frontline ED staff about the recognition of sepsis using these evaluations. The small numbers in our study suggest that antibiotic time did not improve with activation of the SEPSIS KILLS, and identification and activation of the pathway was not associated with improved mortality.

The SIRS criteria have underpinned the principles of detection of bacteraemia in early sepsis recognition pathways that include CEC SEPSIS KILLS. Previous evaluations have demonstrated the tool to be sensitive but poorly specific in the detection of septic patients including those with bacteraemia [19]. However in our population SIRS has demonstrated moderate sensitivity and poor specificity, performing slightly better than in other contemporary studies in this setting [7, 8]. This has become an increasingly recognised phenomenon, which again highlights the diversity of underlying pathophysiology underlying patients with bacteraemia, and can range from gross biochemical and clinical disturbance to minor changes in physiology depending on the time of presentation, underlying comorbidities and pathogen characteristics. SIRS also historically underperformed against more recently developed scoring systems, including qSOFA which has led to its removal from the current SEPSIS-3 definition [20]. Lastly, we found that the criteria could not identify patients at risk of death. This study therefore confirms its lack of value as a clinical prediction tool in sepsis and bacteraemia.

qSOFA was designed as a tool designed to assess severity of illness in sepsis, and initial comparative literature indicated its superiority over its predecessor SIRS in the prediction of mortality and ICU admission [21]. It has however been evaluated as a clinical prediction tool for sepsis and bacteraemia with poor results due to modest sensitivity and specificity [19]. This study confirmed poor sensitivity in clinical diagnosis of sepsis, which we concede is probably driven by its design as an illness severity score rather than a clinical decision making score. The tool was able to predict mortality when it was activated, however it identified few patients in our cohort with bacteraemia and therefore overall was not useful in predicting mortality in this group overall. Similar conclusions have been met, highlighting the inability of qSOFA in identifying mortality risk or likelihood of requiring ICU admission in patients who had severe sepsis, suggesting the need for re-evaluation of whether this tool is clinically useful [12].

In this cohort of patients with sepsis presenting to the emergency department, our study demonstrated also that bacteraemia was associated with a statistically significant increase in hospital length of stay, duration of antibiotics and sepsis-related mortality, when compared to septic patients without bacteraemia. Intensive care unit admission rate and duration was similar in both groups. Contemporary data has demonstrated conflicting evidence when comparing these two cohorts, with some evidence demonstrating higher 30-day mortality and higher and long-term mortality in bacteraemia, while other studies demonstrating no significant difference in mortality or organ dysfunction [22–24]. Considering the heterogeneity of mortality data, we suggest that this should be a focus of further research.

### Limitations

In the design of our study we only controlled our population for age, not other comorbidities which could have significant impact on morbidity and mortality outcomes. Despite patients being randomly selected from a cohort of blood cultures over 2 years, there remains the possibility of selection bias. In addition, our patient population is limited to patients from two moderate sized hospitals, which raises the possibility of reproducibility at other centres. Therefore further studies with a more diverse population are encouraged.

For the benefit of analysis, we used the assumption that patients in the non bacteraemic group never had an episode of bacteraemia. However, we understand that bacteraemia can be episodic, and that taking a blood culture at a single point in time may lead to a false negative result. There are also technical microbiological issues which may yield a truly bacteraemic patient negative, including low volumes of blood inoculated and the presence of organisms that are unable to be cultured.

In the calculation of scores, we used vital signs and clinical information collected within a four-hour period of triage. However, the scoring systems and clinical pathways assessed are designed to be used in real time, therefore this methodology may have led to higher sensitivity scores for the tools then if used as designed. We felt that this was more representative of a true clinical scenario, where there is constant clinical reassessment, especially in a process such as sepsis and bacteraemia where the underlying pathophysiology evolves throughout time.

Lastly this study has not incorporated all current scoring systems in use, such as the New Early Warning score, which has been demonstrated to accurately predict sepsis and mortality when used in conjunction with qSOFA in certain populations. Further studies analysing the sensitivity and specificity of scoring systems when used in combination may demonstrate an improved performance.

## Conclusion

Overall our findings suggest bacteraemia remains clinically important, with higher rates of mortality. We have found that current scoring systems and pathways are not accurate enough to be used as the only tool for diagnosis or mortality prediction. The heterogeneity of presentation of bacteraemia due to underlying physiological differences, comorbid status and variability in the pathophysiology of different pathogens and infective processes likely account for the difficulty in developing a standardised tool for the detection of the bacteraemic and septic process. Further research is encouraged to confirm our findings.

## Supplementary Information

**Additional file 1.** Definitions of Criteria.

## Data Availability

The datasets generated during and/or analysed during the current study are available from the corresponding author on reasonable request.
