# Supplementary material for: Comparison of different sepsis scoring systems and pathways: qSOFA, SIRS, Shapiro criteria and CEC SEPSIS KILLS pathway in bacteraemic and non-bacteraemic patients presenting to the emergency department
Source: BMC Infect Dis. 2022 Jan 22;22:76. doi: 10.1186/s12879-022-07070-6 (PMC8783440; doi:10.1186/s12879-022-07070-6)
Supplement: Supplementary file 1 — Additional file 1. Definitions of Criteria. [file 12879_2022_7070_MOESM1_ESM.docx]

**Additional Material**

S1: Definitions of Criteria

Systemic Inflammatory Response Syndrome (SIRS)

Temperature >38 or <36 ̊C, respiratory rate >20 breaths per minute, heart rate >90 beats per minute, white cell count >12 or <4 or bands >10%

Score ≥2 = meets criteria

Quick Sequential Organ Failure Score (qSOFA)

Altered mental state; respiratory rate ≥22 breaths per minute, systolic blood pressure ≤100mmHg

Score ≥2 = meets criteria

Modified Shapiro Criteria

Major Criteria – suspected endocarditis, temperature ≥39.4 ̊C, indwelling vascular advice

Minor Criteria – age >65, temperature 38.3-39.3, chills, vomiting, systolic blood pressure ≤90mmHg, white cell count >18, bands >5%, platelets <150, creatinine >200mg/dL

≥1 major or ≥2 minor = meets criteria

SEPSIS KILLS Icon and CEC SEPSIS KILLS Pathway

A part of the Cerner FirstNet electronic medical record system is utilised across most EDs in NSW. At triage there is provision to affix to the clinical record a SEPSIS KILLS icon, intended to promote immediate clinical review. The pathway activated at Triage has no specific criteria underlying the application apart from clinical concern. The SEPSIS KILLS icon can only be affixed at triage. The CEC SEPSIS KILLS pathway is a broader document that outlines the key points of recognition and resuscitation of the septic patient. It involves the identification of patients at risk of sepsis using both vital sign derangement and clinical gestalt. This pathway can be commenced by any clinician at any time during the admission.
